# Ultrasmall compact CMOS imaging system for bioluminescence reporter-based live gene expression analysis

**DOI:** 10.1117/1.JBO.26.11.116002

**Published:** 2021-11-03

**Authors:** Joshua Philippe Olorocisimo, Jeric Briones, Kiyotaka Sasagawa, Makito Haruta, Hironari Takehara, Hiroyuki Tashiro, Norihiro Ishida-Kitagawa, Yasumasa Bessho, Jun Ohta

**Affiliations:** a Nara Institute of Science and Technology, Photonics Device Science Laboratory, Division of Materials Science, Graduate School of Science and Technology, Takayama, Ikoma, Nara, Japan; b Nara Institute of Science and Technology, Gene Regulation Research Laboratory, Division of Biological Science, Graduate School of Science and Technology, Takayama, Ikoma, Nara, Japan; c Nara Institute of Science and Technology, Mathematical Informatics Laboratory, Division of Information Science, Takayama, Ikoma, Nara, Japan; d Advanced Telecommunications Research Institute International, Cognitive Mechanisms Laboratories, Kyoto, Japan; e Kyushu University, Department of Health Sciences, Faculty of Medical Sciences, Higashi, Fukuoka, Japan

**Keywords:** biophotonics, micro-CMOS, image sensors, bioluminescence, luciferase, gene expression

## Abstract

**Significance:** Gene expression analysis is an important fundamental area of biomedical research. However, live gene expression imaging has proven challenging due to constraints in conventional optical devices and fluorescent reporters.

**Aim:** Our aim is to develop smaller, more cost-effective, and versatile imaging capabilities compared with conventional devices. Bioluminescence reporter-based gene expression analysis was targeted due to its advantages over fluorescence-based imaging.

**Approach:** We created a small compact imaging system using micro-CMOS image sensors (μCIS). The μCIS model had an improved pixel design and a patterned absorption filter array to detect the low light intensity of bioluminescence.

**Results:** The device demonstrated lower dark current, lower temporal noise, and higher sensitivity compared with previous designs. The filter array enabled us to subtract dark current drift and attain a clearer light signal. These improvements allowed us to measure bioluminescence reporter-based gene expression in living mammalian cells.

**Conclusion:** Using our μCIS system for bioluminescence imaging in the future, the device can be implanted *in vivo* for simultaneous gene expression imaging, behavioral analysis, and optogenetic modulation.

## 1 Introduction

The usual optical devices for bioimaging applications are the photomultiplier tube (PMT), the charged-coupled device (CCD), and the complementary metal–oxide–semiconductor (CMOS) image sensor.^1^ The PMT is the most sensitive of the three; however, it is very costly to make, requires high power, and is usually large and bulky.^2^ The CCD is less noisy than the CMOS, but the readout is slower and requires more power due to the serial transfer of electrons across each pixel.^3^ The advantage of the CMOS is that it is much faster, smaller, and cheaper than the CCD and PMT.

The CMOS fabrication process is commonly used in making integrated circuits. This makes it less expensive to produce in bulk and easily allows different components to be integrated into one chip. Therefore, CMOS devices can be flexible and multifunctional because they can be incorporated into very large-scale integrated (VLSI) circuits, which can be adapted for numerous applications.^4^ It also has the fastest processing time since readout happens in parallel, instead of being passed down serially like in CCD. CMOS image sensors (CIS) are commonly used as camera chips for smartphones since they are easy to integrate and are cost-effective. Finally, another important advantage of the CMOS is the ability to make micro-sized CISs (μCIS), much smaller than conventional CCD or PMT machines. Due to their small size, μCISs can be implanted into biological tissue with very minimal damage, as we have shown previously.^5^^–^^7^ Therefore, μCISs offer a competitive advantage.

We have previously reported the use of these ultrasmall μCIS for fluorescence imaging applications (e.g., ELISA, ${\mathrm{Ca}}^{2+}$ imaging, and FRET).^8^^–^^10^ However, fluorescence imaging requires outside excitation light, which causes high background noise. An effective excitation cut-off filter needs to be designed, which is difficult for small and lensless imaging devices.^11^^,^^12^ The strong excitation light can pass through the filter and add noise to the image. In addition, it can induce autofluorescence in nontarget biological molecules causing false-positives. It can also lead to phototoxicity in a sample especially during long imaging times.^13^ Finally, photobleaching may also occur. These problems can be avoided using bioluminescence imaging (BLI) because it does not need any excitation light and emits a weaker light signal.

BLI offers a higher signal-to-noise ratio since there is less background noise. Small signal changes are easier to detect, which makes bioluminescence more sensitive than fluorescence. Specifically, dynamic gene expression is better imaged using bioluminescence because luciferase has a shorter half-life (3 to 4 h) than conventional fluorescent proteins (25 to 54 h). Changes in gene expression can be more accurately represented by luciferase, since fluorescent proteins take longer to degrade and may not indicate the actual decrease in gene expression. BLI is also better for studies that require longer imaging times because there is less phototoxicity and photobleaching due to its weak light emission. Thus, bioluminescence offers some benefits when imaging gene expression.^14^

Moreover, BLI is advantageous when combined with photomodulation techniques, such as optogenetics. This is because the strong excitation light during fluorescence imaging may activate off-target rhodopsins used in optogenetics, if the excitation and activation spectrum overlaps in some tail-end wavelengths. This can cause lower precision when controlling neural circuits. Since BLI does not require excitation light, then cross-talk can be prevented, which is useful when combining imaging and optogenetics.

**Fig. 1:**
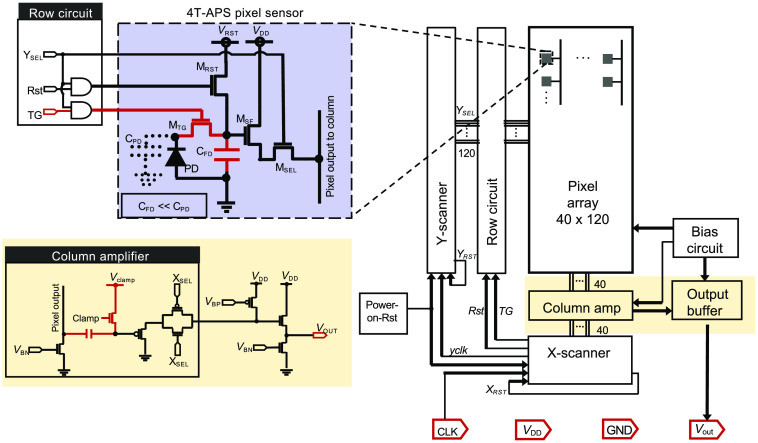
New μCIS architecture highlighted in red is the additional components of the new device compared to our previous models. The device only has four I/O pads to make it more compact. The circuit contains the 4T-APS pixel (violet), column amplifier and output buffer (yellow) circuits. Symbols: voltage (V), capacitor (C), MOSFET (M), reset (RST), select (SEL), source follower (SF), transfer gate (TG), FD, bias P-MOS (BP), bias N-MOS (BN), clock (CLK), voltage drain ($V_{\mathrm{DD}}$), ground (GND), and output (OUT).

However, the use of implantable CIS for BLI has not been previously reported because of the low light intensity emitted by luciferase. Therefore, we developed a new compact device with improved sensitivity by upgrading the pixels from 3T-APS to 4T-APS (Fig. 1). Compared with commercial CISs, the pixel area was made larger, while peripheral components, such as the on-chip analog-to-digital converter (ADC) and lenses were removed. The number of input/output (I/O) pads was also reduced. This enabled the addition of the extra transistor while maintaining the small size of the device.

Another problem for *in vivo* imaging studies is the effect of temperature. The elements in an integrated circuit are affected by changes in temperature. For example, the leakage current of the photodiode (PD) increases and the voltage threshold of the transistors also change.^15^ Noise due to an increase in temperature is the reason that PMT and CCD cameras are placed outside the organism and cooled for bioluminescence detection.^16^ But in our case, the μCMOS device needed to be small for future implantation, so a cooling system was not designed. Instead, we proposed a relatively simple modification on the pixel array. By adding a black absorption filter pattern, we can continuously measure dark current changes due to temperature fluctuation and subtract this from the recording pixels.

The improvements and modifications in our μCIS system enabled us to observe gene expression with luciferase as a bioluminescent reporter under CMV promoter. This new imaging system can be used to measure biological activity without the need for excitation light and cooling equipment. We demonstrate a smaller, simpler, and relatively cheaper method compared to conventional devices. In summary, this study features a compact, lensless, microimaging device for bioluminescence applications.

## 2 Materials and Methods

### 2.1 Device Fabrication

μ-CMOS imaging devices were fabricated for bioluminescence detection and measurement. The device mainly consists of a custom-made CIS chip on a printed circuit board (PCB). It is described further in Sec. 3.

First, the CIS chips were cleaned by submerging them in acetone twice then in isopropanol once, all at 80°C for 5 min in each solution. Then, the chips were placed on silicon glass slides, and a fluoropolymer (CYTOP-M 25%) was applied on the back of the chip for easier detachment later on. The fluoropolymer was dried by heating at 120°C for a few minutes.

Then, an optical adhesive (NOA63, Norland) was applied on the back and around the chip and was then cured under UV light for 90 s. This was to provide more surface area and a level substrate for the device for uniform spin coating later on. Afterward, the device and substrate were carefully detached from the silicon glass slide. The bottom of the device and substrate were attached to another glass slide without silicon, using wax adhesive. It was then spin-coated with a black absorption filter (1:1:1 w/w of red, blue, and green resist mixture). The spin coating sequence was as follows: 3 s slope, 3 s at 500 rpm, 5 s slope, and finally, 20 s at 2000 rpm.

After spin coating, the black absorption filter was alternately removed using a fourth harmonic (266 nm) Nd:YAG laser (TNS Systems LLC) for patterning. The pattern is an alternating two rows of pixels with filter (PWF), then two rows of pixels without filter (POF). The filter on the clock, $V_{\mathrm{DD}}$, ground, and $V_{\text{out}}$ terminal pads were also removed. The laser power was adjusted to find the optimal energy that would remove the filter and not damage the pixels.

Then, the chips were carefully removed from the optical adhesive substrate and attached on a PCB using epoxy, dried at 120°C for 10 min. All the terminal pads on the chips were then wire-bonded onto the PCB. The wires and terminals were then protected with epoxy (Nissin Resin Z-1).

Header pins were then soldered onto the PCB with the image sensor intact. Then, the corresponding wires were connected to the chip. Then, the device was tested whether it was working or not by shining a flashlight on it. Horizontal stripe patterns were observed as expected due to the filter. Pixels were checked if they were damaged. Furthermore, the general resolution of the device was tested using a fiber optic plate (J5734, Hamamatsu) with numbers and symbols.

### 2.2 Device Parameters Testing

After adding the patterned absorption filter and assembling the devices, the (i) dark current, (ii) temporal noise, and (iii) signal sensitivity were tested.

To measure signal sensitivity, a constant LED light source (455, 530, or 625 nm, ThorLabs) was assembled on an optical table and was fitted with an absorptive neutral density filter (ThorLabs) of optical density 3 (0.1% transmittance) or 0.5 (32% transmittance) to reduce the LED’s light intensity. The LED’s current was manipulated using an LED driver (ThorLabs), and the light intensity was measured using a power meter (ThorLabs). Then, the CMOS devices were placed under the light and their raw digital values were recorded.

Next, the temporal noise of the devices was measured. Temporal noise is the fluctuation in signal across time due to factors such as dark current, reset noise, shot noise, and flicker noise. The amount of temporal noise was measured by getting the standard deviation of 128 consecutive frames in each device while in pitch dark condition. Longer exposure times are needed for the detection of low light levels; however, the amount of temporal noise also increases with longer exposure time. To take this into account, the noise of each device was measured at increasing frame/exposure times. This was indirectly increased by lengthening the pixel row settling time, which was the waiting time for the clock reset signal in each row.

Since dark current is one major contributor to temporal noise, it was also measured. To measure this, the average digital pixel values of each device in dark condition were also recorded.

Finally, to measure the signal-to-noise ratio, the average difference between PWF and POF was computed. That is, since PWF block any incoming light signal, whatever value registered here must be due to noise. Then, this value was subtracted to POF to ensure that only the signal from light is measured. A larger difference between the two would mean that there’s a higher signal-noise ratio since the amount of noise is lower compared with the signal only.

### 2.3 CMV Transfection and Luciferase Assay

CMV promoter sequence was subcloned to the multiple cloning site upstream of luc+ in pGL3-basic vector (Promega) to construct pGL3-CMV. Then, $1\times 10^{6}$ HEK293T cells were inoculated with 2 mL of Dulbecco’s modified eagle medium (4.5  g/mL glucose) supplemented with 10% fetal bovine serum (Sigma Aldrich), 100  units/mL penicillin, and 100  g/mL streptomycin (Nacalai Tesque) in a 35-mm tissue culture dish (Thermo Scientific Nunc) and incubated overnight. 2.4 g of pGL3-CMV vector and 7.2 L of 1  μg/mL linear form of polyethyleneimine (Polyscience) were mixed with 100 L of PBS, followed by 20 min incubation at room temperature. Plasmid-polyethyleneimine solutions were added to the culture medium of HEK293T cells. Five hours after incubation, cells were washed with 1 mL of PBS twice and cultured for additional 48 h. Bioluminescence signals derived from luciferase were generated by exchanging medium containing 100 M D-luciferin (Nacalai Tesque).^17^ The signal was detected by placing the CMOS microimaging device below the dish.

### 2.4 Data Analysis

Image data were recorded using custom-made software (CIS_NAIST) and then analyzed using MATLAB (Mathworks). Custom-made codes were written to extract the data from RAW files and convert them into matrix values containing three dimensions (two-dimensional pixel array across time). The values were subtracted from the background measured via fixed pattern noise (FPN) cancellation done in the dark before putting the sample. Then, the pixel array data were trimmed by removing the borders and columns with defective pixels. Afterward, the pixel array was separated into PWF and POF. Frame number was converted into time by dividing the frame number by the average frame rate.

After, the average PWF and POF were plotted per frame. Then, an elementwise subtraction was made between the matrices of PWF and POF. The average per frame was graphed, and the values were normalized by dividing by the total range of values in the data set.

**Fig. 2:**
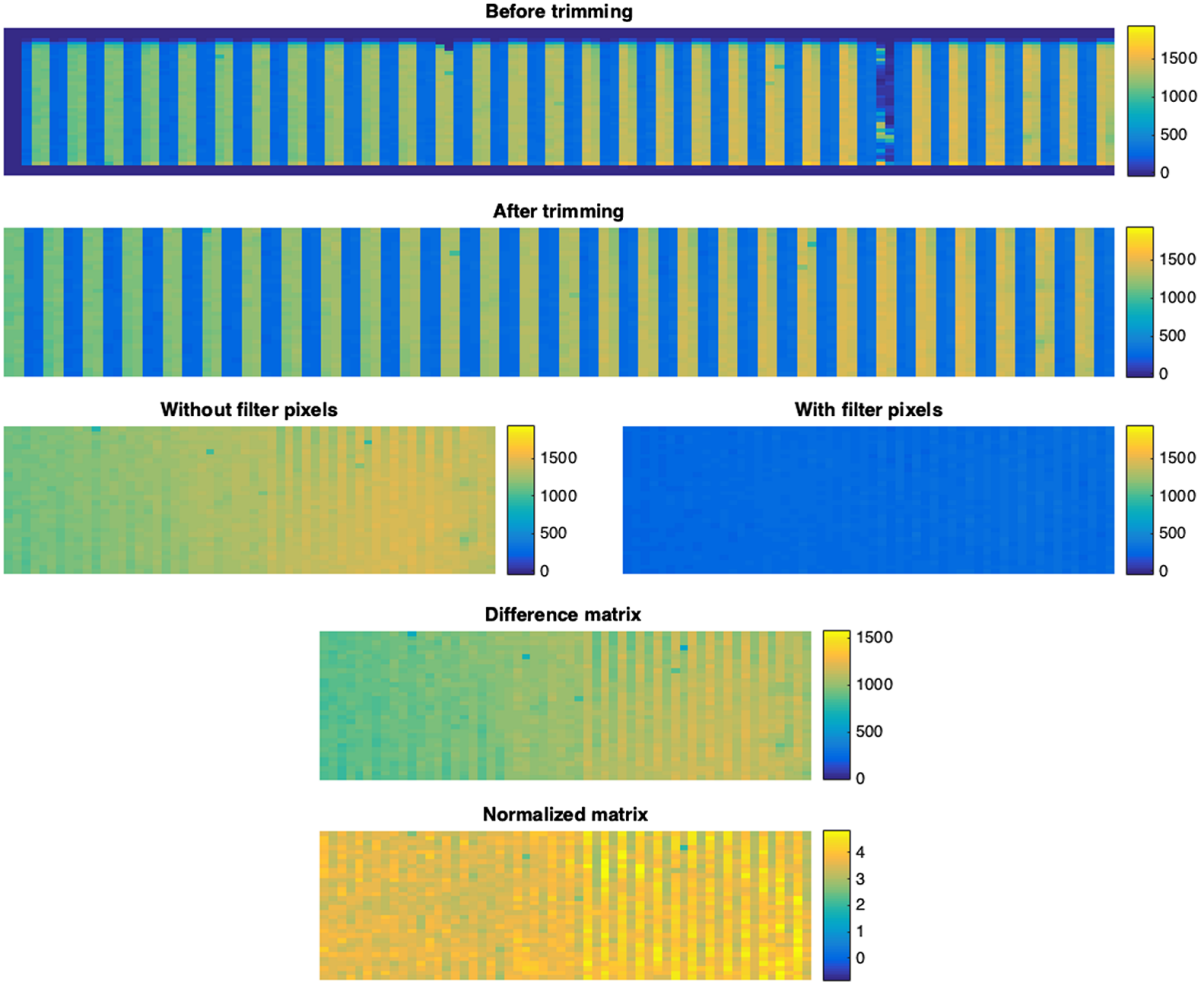
Sample data extraction and analysis. Sample of one frame where digital values are visualized using heatmap from blue (low) to yellow (high). Top to bottom: Matrix before trimming matrix [44×121 digital values], matrix after trimming [32×112], POF matrix (left) and PWF matrix (right) [32×56 for both], difference between with and without filter matrix [32×56], normalized by the dynamic range of the device [32×56].

Figure 2 shows a sample frame extracted from MATLAB. Then, borders and damaged pixels are removed from the pixel array. Afterward, the PWF are separated from POF. Then, the difference and normalized matrices are shown.

The total image of the bioluminescence recording was computed by getting the average of the difference matrix of all frames. Then, the image was deconvolved using blind deconvolution, and then normalized to the range.

## 3 Results

### 3.1 μCISs Were Successfully Assembled and Modified

Two kinds of CIS chips were designed and manufactured: the 3T-APS using 0.35-m standard CMOS process and the 4T-APS using 0.18-m CIS process. These chips were then assembled to create the previous device model^5^^,^^6^^,^^8^^–^^12^ and the new device model featured in this study. The devices were modified and tested in this study to detect bioluminescence signal and image gene expression.

**Fig. 3:**
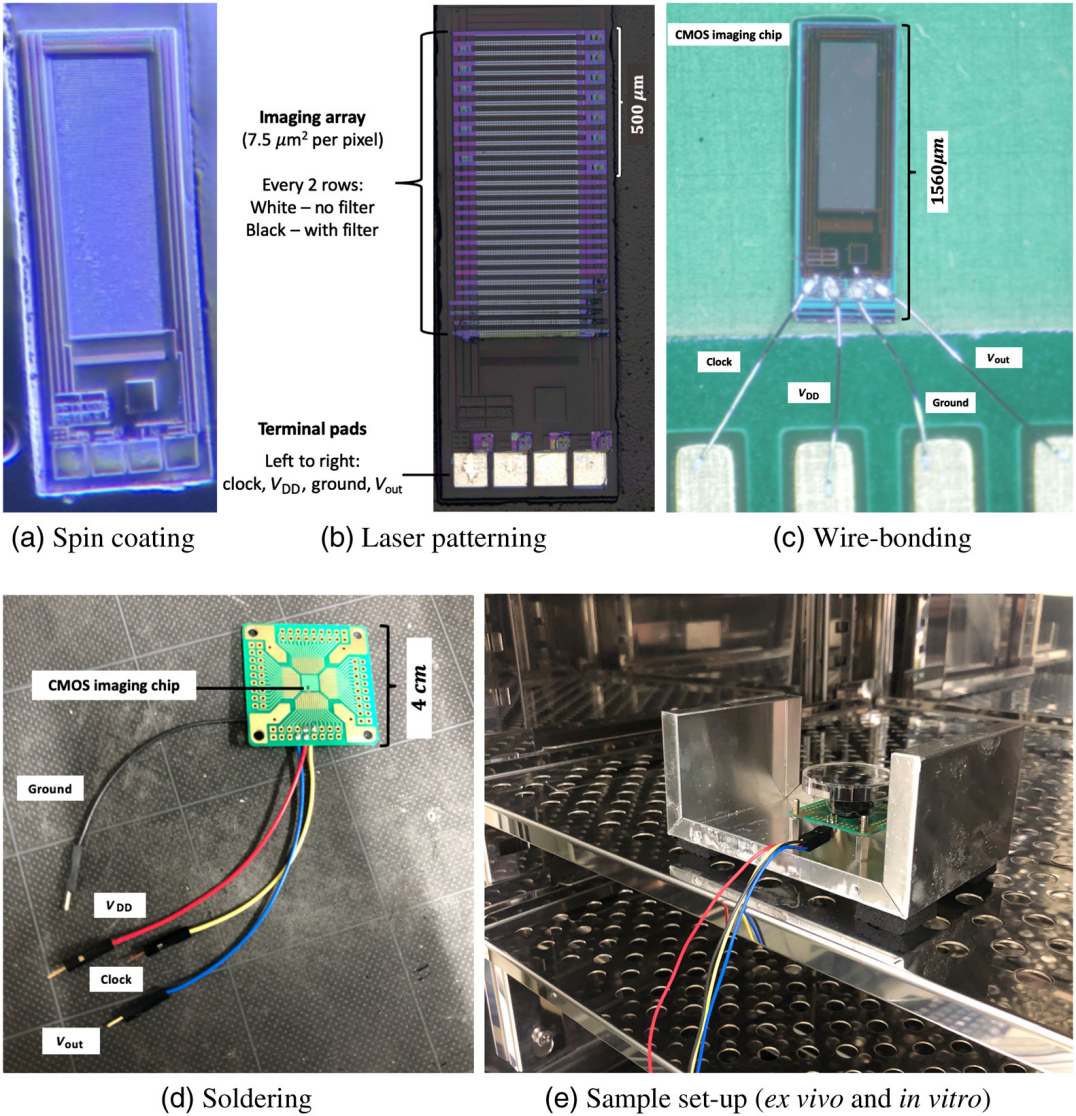
Device assembly and patterning. Sample process for device making. (a) Spin coating of black absorption filter. (b) Patterned removal of filter using Nd:YAG laser. (c) Connecting the device to the PCB (green) using wire-bonding machine. (d) Soldering of copper wires for connecting to the CPU. (e) Sample set-up with plastic-bottom culture dish (black) on top of image sensor encased in a light-tight box (silver; cover not shown) inside an incubator.

First, a black absorption filter was added onto the sensor pixel array by spin coating [Fig. 3(a)]. Then, a Nd:YAG laser was used to etch and design an alternating pattern of PWF and POF [Fig. 3(b)]. This enabled PWF to block any light signal and measure only device noise. Meanwhile, POF measure light signal with device noise. By subtracting PWF to POF, then the purified light signal is attained.

Unlike other conventional devices, our total device size is smaller but the pixel area is larger. The CIS chips were 450  m×1560  m in size, and each pixel was $7.5\;m^{2}$. The on-chip ADC was removed and lenses were not used. In addition, the number of input–output wires was reduced to four terminals (clock, $V_{\mathrm{DD}}$, ground, $V_{\text{out}}$). This makes the devices very small and compact, which is suitable for *in vivo* implantation.

For brain slice culture (*ex vivo*) and cell culture (*in vitro*) setup, the culture dish must rest on a substrate. Therefore, the chips were attached and wire-bonded to a PCB around 4  cm×4  cm in size [Fig. 3(c)]. Then, external wires were soldered on the PCB to connect to the computer [Fig. 3(d)]. Finally, a culture dish can be put on top of the PCB with the chip and encased in a light-tight box placed inside an incubator at controlled temperature and ${\mathrm{CO}}_{2}$ levels [Fig. 3(e)].

Optimization of device fabrication process was also done. It was important to have an even and flat coating of black absorption filter on the pixels so that the filter thickness is constant for all. This was done using an adhesive substrate around the chip to provide more surface area during spin coating. Furthermore, the usage of low Nd:YAG laser intensity for patterning was needed to reduce pixel damage.

Overall, two devices were successfully assembled and fabricated. These are the 3T-APS device (previous model) and the 4T-APS device with patterned filter arrays (new model). These were then compared for their imaging performance.

### 3.2 New Device Model Exhibited Lower Noise and Higher Sensitivity

**Fig. 4:**
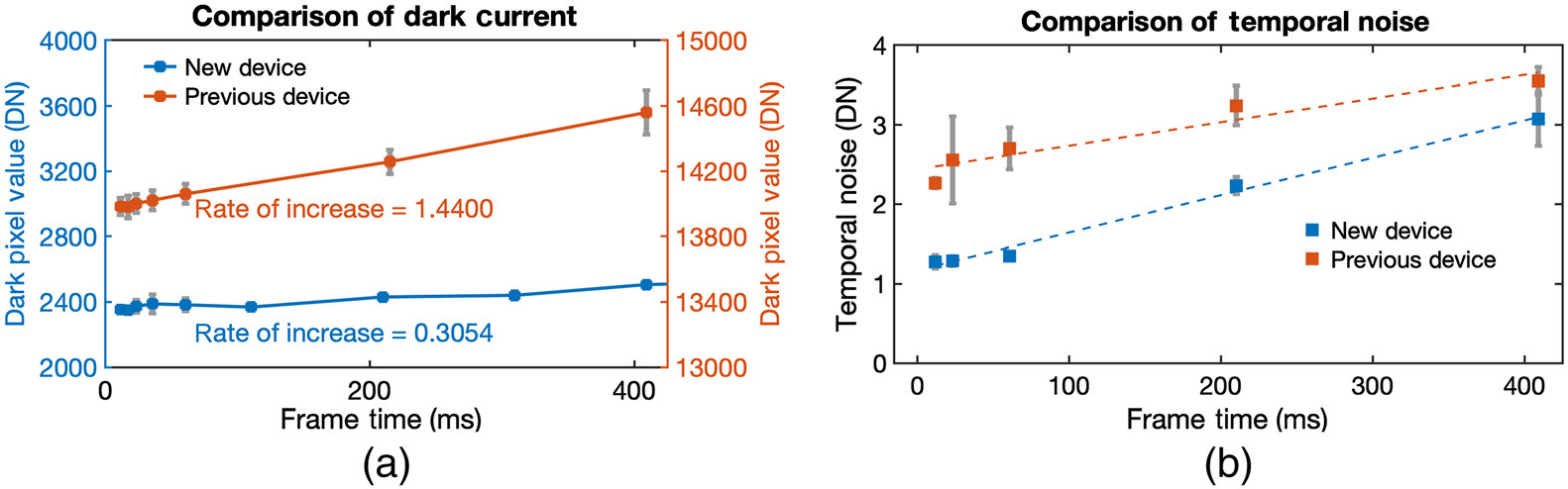
Noise measurement comparison. (a) The dark current and (b) temporal noise of the new (blue) and previous (orange) devices were measured. (a) The y axis of the new device (left axis) and previous device (right axis) both have a range of 2000; however, the starting value in the previous device is much higher. The slope of the line is presented to show the rate of noise increase. The new device showed lower dark current. (b) The standard deviation across 128 consecutive frames under constant light signal was measured. The linear trendline is presented. The new device showed lower temporal noise. The pixel values are in units of DN converted by a 14-bit ADC.

After device fabrication, the noise and sensitivity of the devices were tested using a constant artificial light source (LED light, ThorLabs). Testing was done using 530 nm wavelength (green LED) since this was closest to our bioluminescent model (FLuc=560  nm). First, the dark current of each device model was measured. Dark current is the current generated despite having no actual light signal. To measure this, the signal output from the device was recorded in pitch dark conditions. A longer frame time is needed to increase the accumulation of signal during low-light situations such as in BLI. Therefore, the dark current in various frame times was tested [Fig. 4(a)].

It can be seen that the new device had a lower amount of dark current compared with the previous device. Furthermore, despite the increase of device frame time, the rate of noise increase (i.e., slope) in the new device was only 0.3054. On the other hand, the rate of noise increase of the previous device was 1.4400 as the device frame time was increased. Therefore, the new device exhibited less dark current and lower rate of noise increase compared with the previous device.

Next, after measuring the dark current of the devices, the deviation between consecutive frames given a constant light signal was measured. This standard deviation between 128 consecutive frames was termed as temporal noise. Similar to dark current, temporal noise is proportional to the frame time of the device [Fig. 4(b)].

It can be observed that the new device had a lower trend of temporal noise compared with the previous device. Therefore, the new device provides a more consistent or stable reading across frames even when increasing the frame imaging time.

**Fig. 5:**
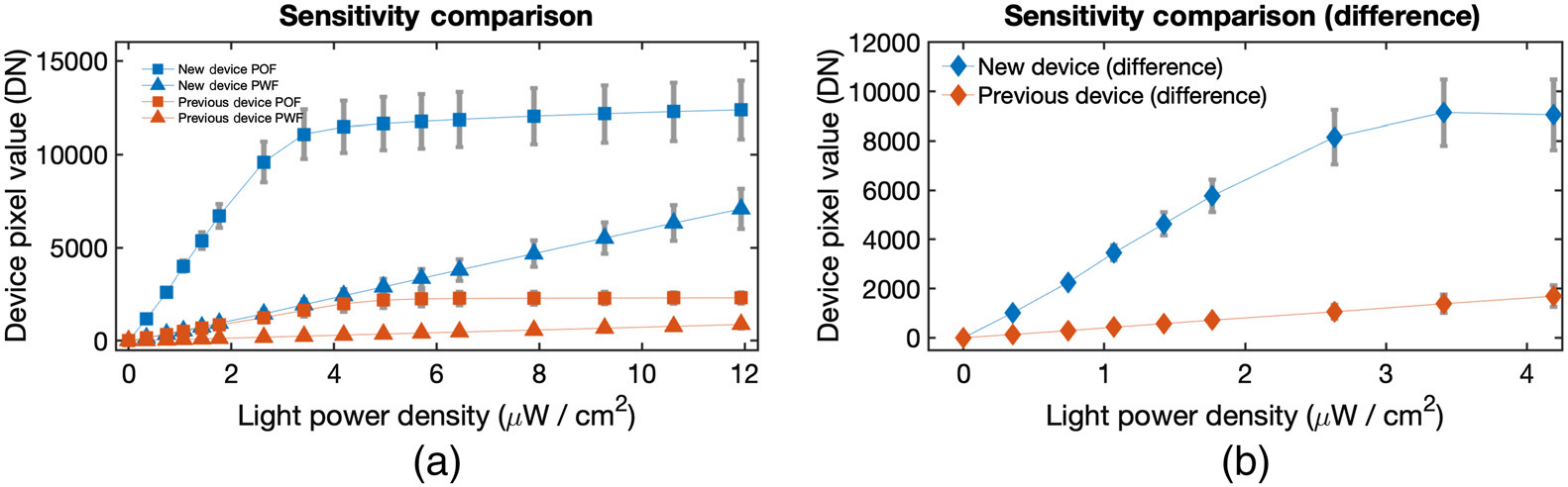
Device sensitivity comparison. The sensitivity of new (blue) and previous (orange) devices was measured. (a) Pixels with black absorption filter (PWF, triangle points) and without filter (POF, square points) of each device were graphed separately. (b) These were then subtracted to get a cleaner signal, and the x axis range was shortened to highlight low light situation. The new device showed higher pixel output value and steeper slope than the previous device. Frame time was 11.82 ms to get base sensitivity.

After the analysis of noise, the sensitivity of the devices was measured. This was done by measuring the pixel output values of the devices across different light intensities. The frame time used for the test was set at the minimum/shortest possible to get the base sensitivity. In a single device, the PWF and POF were graphed separately to differentiate noise recording versus signal plus noise recording [Fig. 5(a)]. These were then subtracted to extract pure signal recording [Fig. 5(b)].

The results show that the output pixel value from the new device is much higher than the previous device. More importantly, the rate of change in output value (i.e., slope) is higher in the new device despite small changes in light intensity/power density. Based on the linear slope, $1\;\mu W/{\mathrm{cm}}^{2}$ of light corresponded to 405.5 digital number (DN) pixel value with an offset of −6.41 DN in the previous device. On the other hand, the new device corresponded to a 3304.3-DN pixel value with an offset of −93.32 DN. This means that the new device was more sensitive to changes in light signal and was also better at detecting low light signals.

The saturation point of the devices can also be observed in the graph wherein the new device slightly saturates at around 3 to $4\;\mu W/{\mathrm{cm}}^{2}$, while the previous device saturates at around $5\;\mu W/{\mathrm{cm}}^{2}$. The new device saturates sooner due to its increased sensitivity. However, luciferase has a power density of $0.1\;\mu W/{\mathrm{cm}}^{2}$ so saturation is not a concern.^18^ Low light situation is more relevant for BLI and was highlighted in Fig. 4(b).

Overall, the new CMOS device model showed superior performance because of its lower dark current, lower temporal noise, and higher sensitivity. Therefore, the new device may be better suited for BLI.

### 3.3 Improved Device Enabled Measurement and Imaging of Bioluminescence In Vitro

To test whether the newly designed device can detect bioluminescence, HEK293T cells were transfected with CMV-driven firefly luciferase (CMV-FLuc) using the PEI method. This would yield a constant and strong signal due to the constitutively active CMV promoter.

**Fig. 6:**
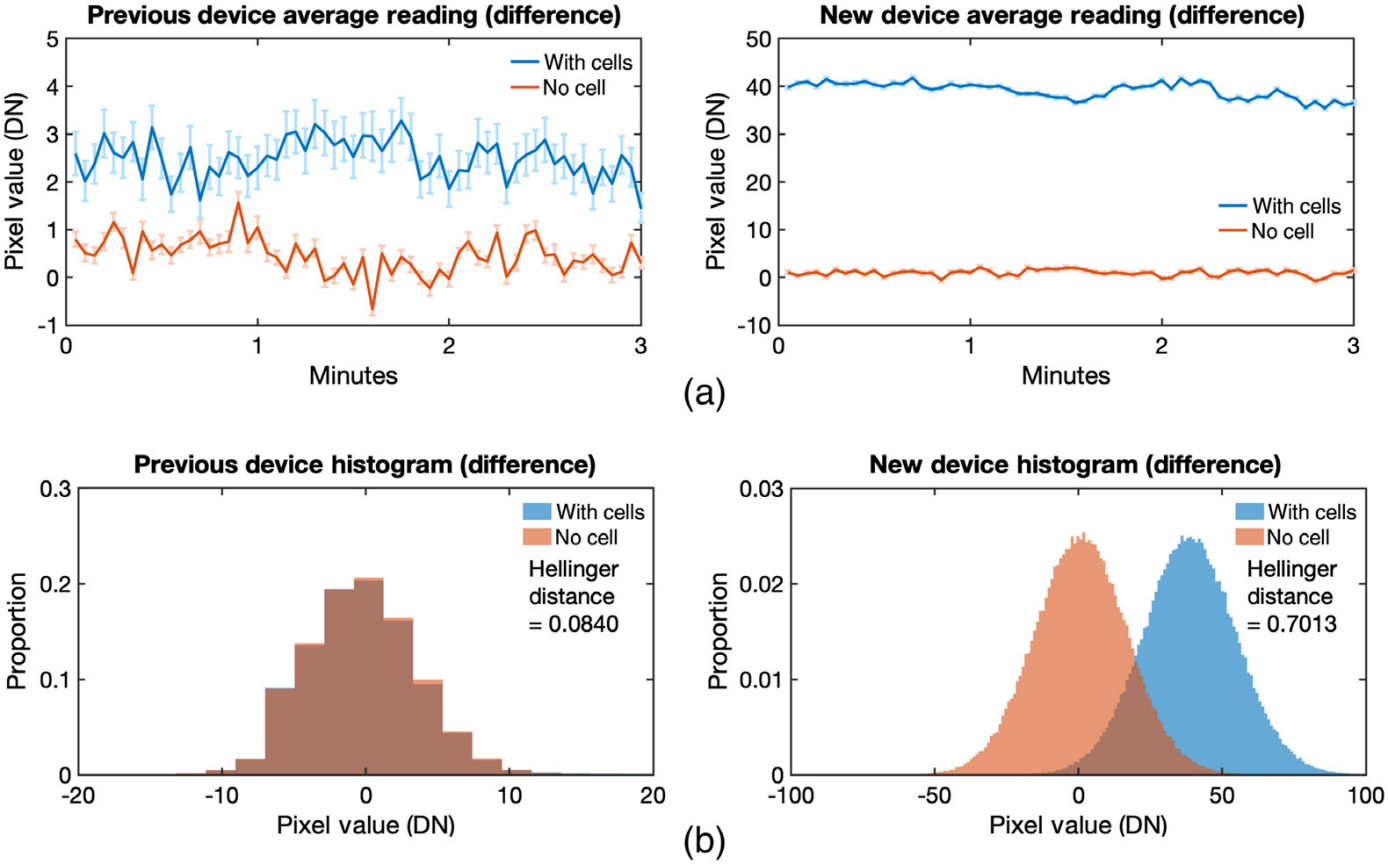
CMV-FLuc BLI of HEKT cells using previous and new devices. (a) Graphs of the light intensity signal are presented either without cells (orange) or with cells (blue). It can be seen that the new device had a higher difference between with and without cells, while the previous device only detected a small signal. (b) Histogram values show the distribution between with and without cell is not so different in the previous device. Quantitatively, the two distributions have a Hellinger distance of 0.08. On the other hand, the new device values show a larger divergence with a Hellinger distance of 0.70. Frame time was 3 s to get highest sensitivity.

Both previous and new devices were tested. First, no cells or dish was placed on the device to measure the background. Then after a few minutes, the HEKT cells were placed on the device. The values of the PWF were subtracted to the POF (difference). The fastest frame rate achievable by the device is 84.5 fps or a frame time of 11.82 ms; however, this setting did not yield any BLI result. To increase sensitivity for BLI, the frame time was extended to 3 s or 0.3 fps, which was the longest capability of the system. As observed, the previous device yielded only a small signal for the set-up with cells compared with no cells [Fig. 6(a)]. However, when the new device was used for imaging, a larger difference in signal between with and without the cells was observed. The values of each pixel in all frames were plotted in a histogram [Fig. 6(a)]. The histogram showed no distinction between with and without cells for the previous device. On the other hand, a divergence of the data sets in the new device was observed. This means that the new device was able to differentiate the set-up with CMV-FLuc signal from the set-up without any signal.

**Fig. 7:**
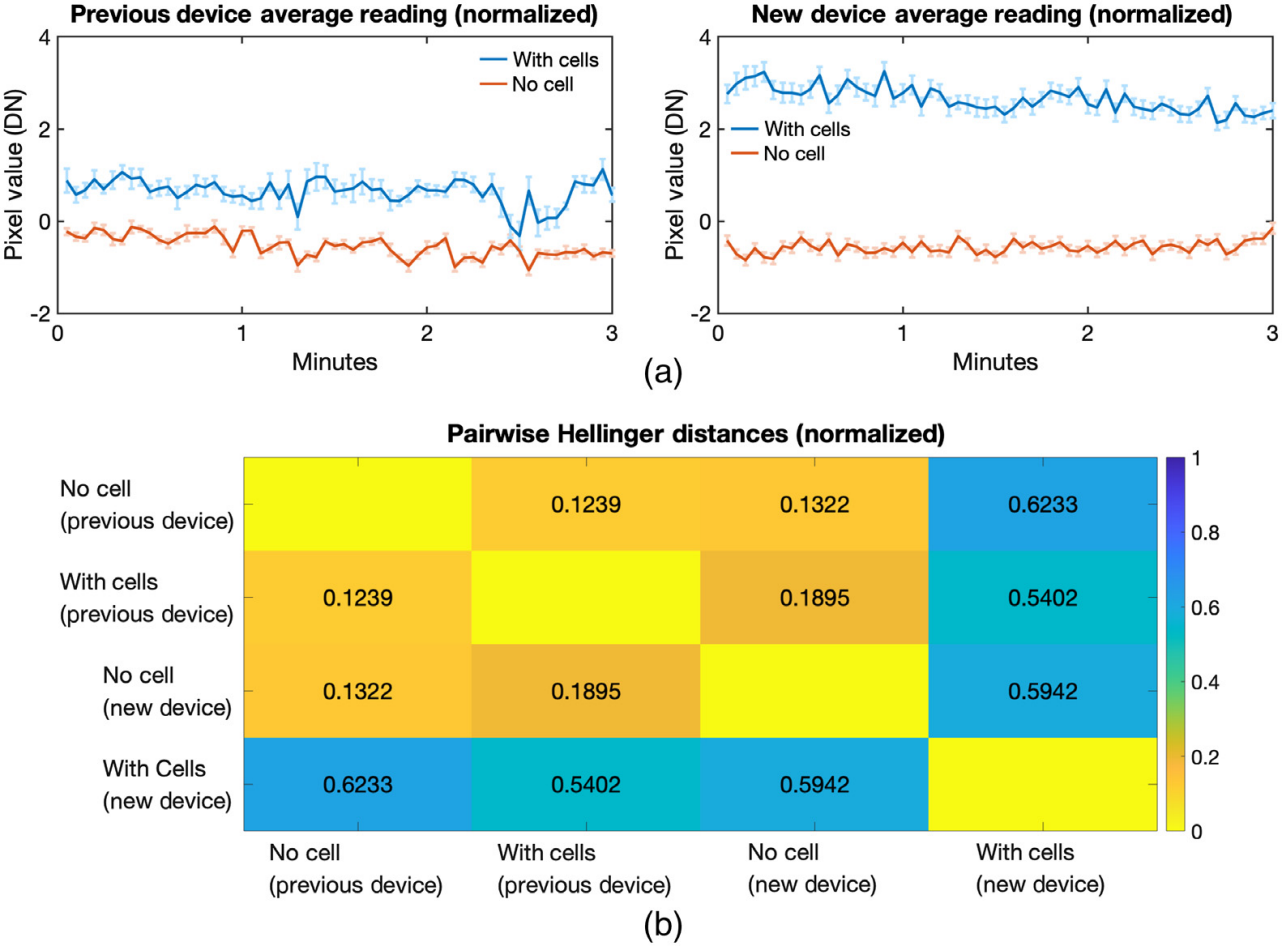
Normalized BLI comparing previous and new devices. (a) Values were divided by their respective range of each device to enable comparison between the two. The new device was able to detect a larger bioluminescent signal than the previous device. (b) Pairwise Hellinger distances were computed between each dataset. This shows the amount of divergence when comparing the data. A value of 1 means the datasets have very different values and distributions while 0 means they are similar.

Then, the result was normalized to the range for comparison between devices [Fig. 7(a)]. A signal of around three was detected for the new device with cells, but only a signal of one was detected by the previous device. This shows that the new device detected three times increase in signal compared with baseline when the dish with bioluminescent cells was placed. The Hellinger distance between the different datasets was computed to measure the f-divergence of the distributions [Fig. 7(b)]. This measures the dissimilarity between two distributions of data and is used for applications such as image analysis since it is more robust and less affected by outliers, unlike univariate hypothesis tests.^19^^–^^21^ Using this measure, it can be seen that the new device with cells had high divergence from the rest of the set-ups (blue-green to blue color). The imaging of cells using the new device yielded a Hellinger distance of above 0.5 when compared with other set-ups. On the other hand, the rest had a Hellinger distance of below 0.2 (yellow to orange) when compared with each other. This indicates that the data from the new device when imaging bioluminescent cells were different from the other set-ups and supports that a signal was detected. Furthermore, comparison of both devices with cells showed a Hellinger distance of 0.54, indicating a difference between new and previous devices during cell recording. This may be attributed to the fact that the new device was more sensitive and detected a larger signal than the previous device.

**Fig. 8:**
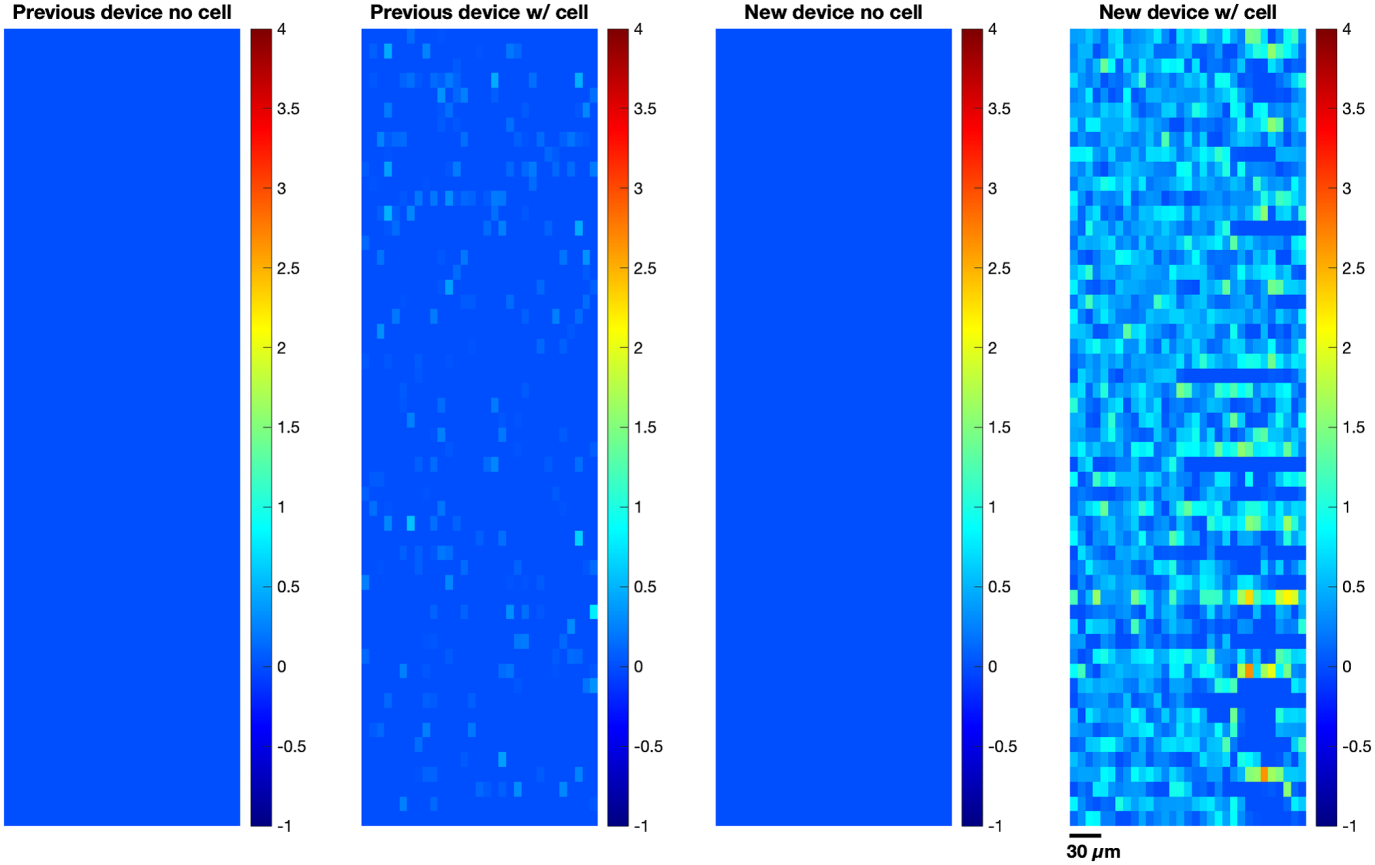
Total average image of bioluminescence recording. Sample image of the average of all frames in the recording. The average was computed from the difference matrix, then deconvolved and normalized by the range. Bright spots appeared upon placing the cells in both old and new devices. However, the new device detected higher number and higher intensity of bioluminescent signal.

Finally, because our system is an image sensor, we can also see spatial differences in bioluminescence signal, unlike conventional photodetectors. An average image of the bioluminescence recording is presented (Fig. 8). It can be seen that upon addition of confluent cells, both the previous and new device had changes in the intensity of their pixels. However, the new device could detect a higher number and brighter intensity of confluent cells emitting bioluminescence signals. A common plastic-bottom dish with a thickness of 0.8 mm was used for imaging to demonstrate the applicability of our device in usual cell culture laboratories. In addition, our device was lensless to allow for *in vivo* implantation in future applications. These conditions reduced the resolution of the device, but the improved sensitivity of our microimaging device showed both spatial and temporal differences in bioluminescence signal even without additional optics. All in all, the new and improved device was able to detect bioluminescence signal better than the previous device. This enabled us to record and image spatiotemporal gene expression of CMV-FLuc in mammalian cells.

## 4 Discussion

### 4.1 Improved Micro-CMOS Device

As presented in Sec. 3, an improved device was attained using the 4T-APS pixel architecture and black absorption filter pattern. There are several important advantages of 4T-APS over the 3T-APS pixels (Fig. 1). One main advantage is the photodetection (photon-to-electron) and photoconversion (electron-to-voltage) regions of the 4T-APS are separated. This is possible due to its additional components: the floating diffusion (FD) for charge storage and the transfer gate ($M_{\mathrm{TG}}$) for controlled access to FD. The $M_{\mathrm{TG}}$ is the additional transistor in the 4T-APS. The PD serves as the photodetection region, whereas the FD serves as the photoconversion region. The light signal gathered by the PD is transferred to the FD. The FD has a smaller size and capacitance than PD, which leads to a larger voltage difference during readout and finally a higher signal reading. This is one reason why 4T-APS is more sensitive than 3T-APS. The PD cannot have a small capacitance because a large surface area is needed for capturing light. Therefore, having an FD with smaller capacitance is integral. Furthermore, the separation of PD and FD allows for the measurement of reset noise via correlated double sampling (CDS). Pixel reset must occur to prepare for the next frame. However, some charges remain in the pixel after reset which adds noise to the sample. To remove this reset noise, the FD resamples from the PD right after reset, and this is subtracted from the signal. In total, the sampling occurs twice once during signal readout and another after reset thus, it is called CDS.^22^

Another advantage is that 4T-APS structures have pinned PDs, meaning that the PDs are buried deeper into the substrate to prevent traps caused by the interface between the silicon dioxide layer and silicon layer. In addition, the pinned PD is completely depleted, and thus all of the accumulated charges in the pinned PD can be transferred into FD. This pinned PD structure reduces dark current and allows for easy signal transfer to the FD.^23^ However, one drawback of having the 4T-APS design is that it is more difficult to manufacture. In addition, if there is an incomplete PD-to-FD charge transfer, a lag may occur. Despite these minor drawbacks, our more compact 4T-APS CIS displayed better performance than the previous 3T-APS model.

Additional improvements were implemented in our device for BLI. The new device was fabricated using a 0.18-m CIS process. It has a higher sensitivity due to higher quantum efficiency and lower dark current compared with the previous device that used 0.35-m standard CMOS process. Our pixel and PD area were larger than commercial devices that also use 0.18-m fabrication process because we wanted to increase sensitivity. But to keep the device small and implantable, we removed other components such as the on-chip ADC and lens. Furthermore, the number of I/O pads were reduced by recycling the clock signal for both the row and column. Given these modifications, our device has a low temporal dark noise (1 to 3 DN) and high sensitivity similar to commercialized miniature CMOS devices,^24^ but our device is more compact and lensless which is not commonly found in the market.

Since the target application is for biomedical imaging, additional noise sources are present due to temperature fluctuation in biological systems and its environment. To take this into account, the black absorption filter was applied on some pixels. This filter blocked any incoming light signal and allowed for the measurement of dark current and other noise sources common to all pixels. This can then be subtracted to the other pixels exposed to light. By having such a structure, the actual signal readings from the device can be separated from noise due to temperature changes.

### 4.2 Bioluminescence Imaging Applications of the CMOS Device

CMV-driven firefly luciferase BLI was successfully performed using our new device. This is important because detecting bioluminescence has a wide array of applications. For example, a bioluminescence lab-on-chip for luminometry applications such as pyrosequencing has been designed.^25^ A 0.18-m CMOS process was also used to fabricate the device, but with fewer pixels—8×16  pixel array—compared with our 40×120  pixel array. Thus, their device sensitivity was higher, but our resolution was better for cell imaging. In addition, CIS in smartphone cameras was used to detect luminescence for bacterial monitoring of *Pseudomonas fluorescens* M3A.^26^ The set-up involved a specialized chamber that would collect light and direct it toward the CIS. The exposure time used was up to 60 s, which allowed for longer signal accumulation. Then, bioluminescent bacteria of at least $7.9\times 10^{6}\;\mathrm{CFU}/\mathrm{mL}$ were detected. Despite the shorter max exposure time of our μCIS device (3 s), a bioluminescence signal from mammalian HEK293T cells seeded at $0.5\times 10^{6}\text{ cells}/\mathrm{mL}$ was still detected. Thus, incorporating our new μCIS devices may yield better resolution and sensitivity in the aforementioned applications.

Given the characteristics of our device, bioluminescence was detected using a strong active CMV promoter in mammalian HEK293T cells. Although signal intensity of CMV-driven expression varies across cell types, it was reported that CMV had the strongest intensity in HEK293T cells compared with other constitutive promoters such as SV40, CAGG, TRE, and UBC.^27^ Furthermore, due to the small surface area of HEK293T cells, a higher density of cells can be achieved in confluent conditions. Also, a high amount of plasmid can be transfected into the cells, thereby increasing the copy of luciferase genes.^28^ The combined increased performance of our new device and strong expression of CMV-driven luciferase allowed us to visualize bioluminescence activity in cells. Hence, if natural or endogenous gene expression shall be studied *in vivo*, the use of brighter luciferases, such as NanoLuc or AkaLuc, would be more suitable than the conventional firefly luciferase used in this study.^29^

Notably, this study serves as a proof-of-concept for *in vivo* BLI of gene expression. Due to the small size of our imaging chips, it can be implanted into freely moving animals. This was demonstrated previously using fluorescent reporters.^6^^,^^7^^,^^30^ However, as mentioned earlier, fluorescence imaging has some drawbacks and so we developed a new device for bioluminescence instead. This new device still follows the same size as our previous studies. Similarly, the device can also be coated with Parylene-C to protect the device and make it biocompatible.^31^ Therefore, the device presented in this study can be used for gene expression analysis in freely behaving animals. Measuring gene expression in freely moving conditions is important to understand the relationship between genes and behavior. For example, understanding the relationship of clock genes with behavioral rhythms still remains a major challenge in the field of chronobiology.^32^^–^^34^

Few current techniques can image and measure real-time bioluminescence gene expression in freely behaving animals. Some studies made use of implanted optical fibers; however, this was generally connected to a PMT and so was difficult to set-up and move around.^35^^–^^37^ Furthermore, a single PMT is more expensive than multiple CMOS devices. Another study made use of cooled EM-CCD cameras to image luciferase-reporter-based gene expression noninvasively.^16^ The drawback of this however is that the luminescence can only be visualized in a general brain region. Also, the experiment must be done in a very dark box which limits the possible behavioral tests that can be done in tandem with imaging. To the best of our knowledge, none of the previous methods could image *in vivo* gene expression, conduct behavioral tests, and do optogenetic manipulation of neurons all at the same time.

That is why an implantable μCIS would be advantageous. It can allow the animal to move freely in a semilighted room, which may allow for more behavioral tests. It is also smaller and more portable than other devices. In our case, the CMOS device is only around 1 mm in length, which makes it possible to implant into the brain with minimal damage. Furthermore, a guide cannula can be inserted to allow for administration of chemical substrates. Our CMOS devices can also be incorporated with μLEDs, which allow for photoexcitation of light-sensitive proteins such as rhodopsins needed for optogenetic manipulation of neurons.^5^^,^^9^^,^^30^

### 4.3 Further Developments for In Vivo Bioluminescence Imaging

To image clearer signals during *in vivo* monitoring of gene expression in a freely moving animal, device sensitivity must be further increased. One of the biggest factors that reduce the detection limit is noise. Noise can occur either at the pixel-level circuit or column-level circuits such as in ADC and amplifiers.^38^ Furthermore, noise can be classified into two kinds: temporal noise and FPN. FPN does not change over time and is caused by physical structure defects on the imaging chip, such as uneven pixel fabrication. Since it is time-invariant, FPN is easy to cancel by measuring the pixel values in a completely dark situation. This was done in our case by measuring around 250 frames in pitch dark condition and using this as the baseline value. This can also be done *in vivo* by turning off outside light and recording before administration of the luciferin substrate. An additional way to cancel FPN noise is through CDS.^39^

Temporal noise is more difficult to remove, because it varies across time and is therefore different in each frame. Sources of this noise include: thermal noise, reset noise, 1/f noise, photon shot noise, and random telegram signal noise. One prominent noise source in low-light situations is the dark shot noise. This is proportional to the square root of exposure time and dark current, where dark current is time-variant and temperature-dependent.^22^ These factors are important to measure because a high exposure time is needed for low-light BLI, and temperature fluctuates in biological systems such as *in vivo* or upon adding chemical substrates *in vitro*. This is why the black absorption filter pattern was important.

The black filter enabled us to control for the effects of dark current drift caused by temperature changes. By having some pixels measure dark noise instead of light signal, it allowed us to measure and subtract the temperature-dependent background offset without the need for a cooling system. Therefore, our device was still small enough to be implantable. Other modifications can also account or reduce such temperature dependent noise in CMOS devices;^40^^,^^41^ however, our method was simpler. The trade-off is blocking some pixels from receiving signal, thereby reducing resolution and imaging area. Nonetheless, other filter patterns can be used, such as a mosaic or tiled pattern, to reduce the number of pixels blocked. Since improved sensitivity to low light was the main target, our proposed method is still applicable.

In addition to noise inside the device, outside noise factors can also be considered. An example is the noise susceptibility of the copper wires connecting our device to the circuit board. This is very prone to noise because it carries an analog signal that is only converted to a digital signal in the circuit board. Analog signal is more prone to noise, and one way to solve this is to put ADCs into the pixel or chip architecture itself, as was done by Eltoukhy et al.^25^ However, this can lead to a larger chip, more heat, and prohibit *in vivo* implantation.

Aside from hardware improvements, the use of computational techniques and software is also another way to extract signal from noisy data. Kim et al.^26^ made use of a noise reduction ensemble averaging algorithm to filter out noise and amplify the signal. This was done by averaging a group of pixels in a certain circumference to smoothen the image (i.e., circular averaging filter). This resulted in the cancellation of random white noise; however, averaging also reduced signal intensity in the process. To solve this, they accumulated the signal of 10 consecutive frames and iteratively put them on top of each other to extract a brighter luminescence signal. Finally, the signal itself can be improved using brighter luciferases. New bioluminescent proteins are being developed that demonstrate brighter emission, higher stability, and longer wavelengths for better *in vivo* imaging in the future.^42^

## 5 Conclusion

In summary, an improved μCMOS device suitable for brain implantation was developed and tested. The previous 3T-APS pixel architecture was improved to 4T-APS and modified by adding a filter pattern array. The new device showed superior imaging performance as shown by its lower dark current, lower temporal noise, and higher sensitivity. In addition, the black absorption filter pattern allowed us to measure and subtract temperature-dependent dark current drift. Because of these improvements, the new μCIS device was able to detect bioluminescence of CMV-FLuc HEKT cells, while the previous device model could not. To the best of our knowledge, no other published studies have reported the use of μCIS devices to detect CMV-FLuc bioluminescence in mammalian cells.

By implementing improvements in CMOS device fabrication and circuitry, lower noise and higher sensitivity can be achieved. Computational algorithms may also prove beneficial by using statistics and machine learning to remove noise, increase sensitivity, and improve resolution. Finally, integrating this with better biotechnology by developing new biosensors such as brighter luciferases may enable *in vitro* to *in vivo* BLI of gene expression, among other applications.

## Data, Materials, and Code Availability

Codes for the analysis are available at: https://github.com/jercb/micro-cmos.
